# The countdown to type 1 diabetes: when, how and why does the clock start?

**DOI:** 10.1007/s00125-023-05927-2

**Published:** 2023-05-26

**Authors:** Anette-Gabriele Ziegler

**Affiliations:** 1 Institute of Diabetes Research, Helmholtz Munich, German Center for Environmental Health, Munich, Germany; 2 Forschergruppe Diabetes, School of Medicine, Klinikum rechts der Isar, Technical University Munich, Munich, Germany; 3 Forschergruppe Diabetes e.V. at Helmholtz Munich, German Research Center for Environmental Health, Munich, Germany

**Keywords:** Autoimmunity, Beta cell, Childhood, Environmental exposures, Genetic predisposition, Immune response, Inflammation, Islet autoantibodies, Review, Type 1 diabetes

## Abstract

‘The clock to type 1 diabetes has started when islet antibodies are first detected’, commented George Eisenbarth with regard to the pathogenesis of type 1 diabetes. This review focuses on ‘starting the clock’, i.e. the initiation of pre-symptomatic islet autoimmunity/the first appearance of islet autoantibodies. In particular, this review addresses why susceptibility to developing islet autoimmunity is greatest in the first 2 years of life and why beta cells are a frequent target of the immune system during this fertile period. A concept for the development of beta cell autoimmunity in childhood is discussed and three factors are highlighted that contribute to this early predisposition: (1) high beta cell activity and potential vulnerability to stress; (2) high rates of and first exposures to infection; and (3) a heightened immune response, with a propensity for T helper type 1 (Th1) immunity. Arguments are presented that beta cell injury, accompanied by activation of an inflammatory immune response, precedes the initiation of autoimmunity. Finally, the implications for strategies aimed at primary prevention for a world without type 1 diabetes are discussed.

**Graphical Abstract:**

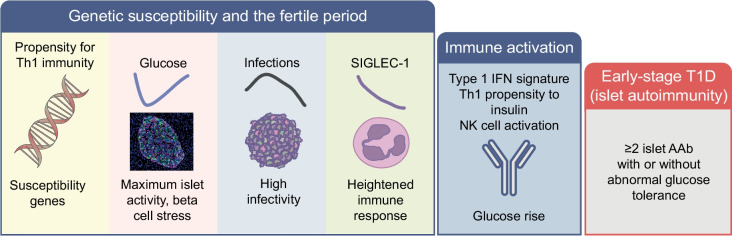

**Supplementary Information:**

The online version contains supplementary material available at 10.1007/s00125-023-05927-2.

## Introduction

The clinical manifestation of type 1 diabetes is preceded by pre-symptomatic islet autoimmunity [1, 2], which is diagnosed by the detection of islet autoantibodies [3]. The presence of more than one islet autoantibody (classified as stage 1 of type 1 diabetes disease) is associated with a high probability of developing clinical, symptomatic diabetes over the following years [4]. This review focuses on the questions of when, how and why islet autoantibodies first arise. It will emphasise fertile periods and outline the combination of factors that favour the development of autoimmunity targeting beta cells. It also describes the metabolic and immunological features that are characteristic of the fertile period, and how these features change prior to the initiation of islet autoimmunity. Potentially relevant environmental exposures during the fertile period are also discussed. Finally, it summarises how the assembled findings can be used to plan future strategies aimed at arresting or reducing the risk of islet autoimmunity.

Data from three longitudinal birth cohorts are considered to characterise the susceptible periods for islet autoimmunity. The BABYDIAB/BABYDIET study [5, 6] commenced in 1989 and has gained over 30 years of prospective follow-up. It is the first birth cohort in diabetes and included 2441 children with a first-degree relative with type 1 diabetes. The Environmental Determinants of Diabetes in the Young (TEDDY) study [7], which commenced in 2004, is an international birth cohort of 8676 children with increased HLA-defined risk for type 1 diabetes recruited from the general population or from families with type 1 diabetes in the USA, Finland, Sweden and Germany. Finally, the Primary Oral Insulin Trial (POInT) [8], which started recruitment in 2018, has enrolled 1050 children with a high polygenic risk score for type 1 diabetes across Belgium, Germany, Poland, Sweden and the UK. POInT is testing whether oral insulin immunotherapy reduces the emergence of islet autoantibodies. All of these three studies are following children for the development of islet autoantibodies and the clinical manifestation of type 1 diabetes.

## Islet autoimmunity: when?

Little, if anything, was known about the timing of autoimmunity in type 1 diabetes when the BABYDIAB study commenced in 1989, and it yielded some surprising results. The first surprise was that several children had already become islet autoantibody positive prior to 1 year of age [9]. The second was that there was a period of peak incidence for islet autoimmunity [10–12]; both BABYDIAB and TEDDY conclusively showed that incidence peaked at 1–1.5 years of age [13, 14]. The peak age was similar between children with a first-degree relative with type 1 diabetes and children from the general population without a close relative with type 1 diabetes [13]. These intriguing data indicate that there is a fertile period early in life with enhanced susceptibility for autoreactivity to beta cell targets. Using landmark modelling, we learned that the risk of developing islet autoantibodies declines exponentially with age [15]. This has practical relevance for screening and consulting families. For example, an infant born to a father with type 1 diabetes has an estimated risk of 7% for developing islet autoantibodies by 6 years of age. If this child remains islet autoantibody negative at 6 years of age, the remaining risk of developing islet autoantibodies over the next 6 years (by 12 years of age) is only 1% [15]. The half-life for this decline in risk is ~3 years [16] during childhood and adolescence, reaching a plateau at ~1%. It should be emphasised that it is likely that islet autoimmunity can develop at any age, and most cases of islet autoimmunity are expected to occur after the age of peak incidence. Nevertheless, besides aiding with the practical aspect of when to screen for risk, the age of peak incidence and the exponential decay in risk with age have important implications for the aetiology and pathogenesis of type 1 diabetes because the genes and/or environmental factors that influence islet autoimmunity must exert their greatest effect at or prior to the age of peak incidence.

## Islet autoimmunity targets: how?

Early autoimmunity is directed against insulin, and memory insulin-reactive T cell responses and IAA have a similar temporal appearance [17–19]. IAA are associated with *HLA-DR4* [20], and most children who develop their first islet autoantibody during the susceptible peak period carry a genotype containing *HLA*-DRB1*04 [21]. In contrast, children with a homozygous *DR3/3* genotype preferentially develop antibodies against GAD, usually later in life [19, 22, 23]. It is unclear why insulin is the key antigen in the early autoimmunity period and why newly emerging autoimmunity to insulin declines with age. It is possible that increasing peripheral tolerance towards an abundant circulating antigen, like insulin, plays a role. This is supported by Daniel and colleagues, who showed that the number of insulin-specific regulatory T cells (Tregs) increases with age and with the duration of autoimmunity [24].

The target epitope and antibody affinity are critical to the relevance of IAA in diabetes [25]. Only children with high-affinity autoantibodies progress to clinical type 1 diabetes, and several antibody assays (electrochemiluminescence [ECL], luciferase immunoprecipitation system [LIPS] assay and radiobinding assay [RBA]) have been developed or adjusted to distinguish between high- and low-affinity responses [26]. High-affinity IAA are associated with HLA-DRB1*04 and a young age at appearance. High-affinity IAA require conservation of human insulin A chain residues 8–13 and react with proinsulin. In contrast, low-affinity IAAs are dependent on the COOH-terminal B chain residues in insulin and seldom bind to proinsulin.

## Role of genetics in autoimmunity

A type 1 diabetes-associated genetic constellation is the first important component of the fertile environment [27]. Before the BABYDIAB study began, it was unclear whether type 1 diabetes-associated genes were responsible for the development of islet autoimmunity or the progression to type 1 diabetes. It was shown that genetic susceptibility is important for initiation of islet autoimmunity and markedly less important for disease progression [21, 22, 28–31]. HLA and non-HLA genes strongly influence the risk of developing islet autoantibodies [32]. The prevalence of multiple islet autoantibodies in children from the general population without a close relative with type 1 diabetes is ~0.31% (95% CI 0.27%, 0.35%) [33]. Children with the HLA genotypes *DR3/4-DQ8*, *DR4/4-DQ8* or *DR3/3* have an average risk of ~5% of developing multiple islet autoantibodies by 6 years of age [34]. Adding non-HLA genes to polygenic risk scores can further stratify the risk and identify newborns in the general population with a 10% risk of developing multiple islet autoantibodies by 6 years of age [32]. The genetic risk changes with age. Moreover, importantly, the influence and risk hierarchy of HLA and *INS* genotypes, which are strongest in the first years of life, are weak or non-existent by 6 years of age [16]. This indicates that a substantial component of the genetic risk for type 1 diabetes involves the risk of developing islet autoimmunity in the fertile age period. This also means that the genetically defined mechanisms of susceptibility operate early in life, and that either genotype-associated functional differences are more pronounced in early life or that ‘co-factors’ for the functional differences conferring susceptibility are missing later in life. Most genes associated with type 1 diabetes are involved in or influence the immune or cellular responses to infection (e.g. *IFIH1*, *TYK2*) [35–37]. For example, HLA class II genes influence the strength of binding between HLA class II molecules on antigen presenting cells and peptide–T cell receptor complexes on T cells, and, hence, thymic selection of Tregs, and may, therefore, confer the greatest susceptibility at a young age, when the thymus is most active [38–40]. It is also possible that some genes influence other factors involved in the pathogenesis of type 1 diabetes. Somewhat unforeseen, but not necessarily unexpected, the susceptible *HLA-DR4* and *INS* genotypes are associated with altered microbiome compositions during childhood [41, 42], whereas *HLA-DR4* is associated with an increased birthweight [43, 44], and the microbiome and birthweight have been reported to be associated with the development of islet autoimmunity and type 1 diabetes [45–50].

## Early life is a fertile period for initiation of islet autoimmunity: why?

### The case for a trio of factors: islet susceptibility/vulnerability, increased exposures and heightened immune responses

#### Islet susceptibility/vulnerability

**Fig. 1:**
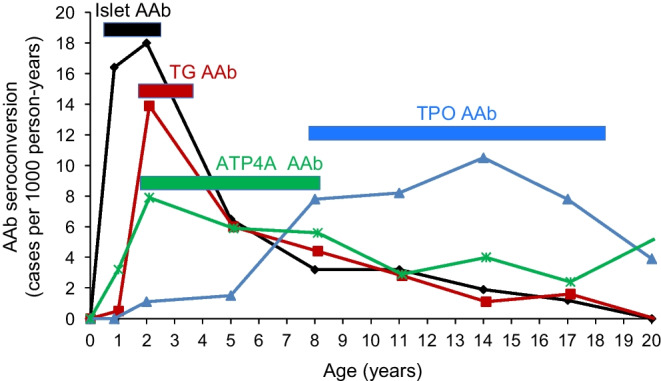
Age-related incidence (cases per 1000 person-years) of autoantibodies (AAb) in individuals with a first-degree relative with type 1 diabetes. Genes predisposing for type 1 diabetes and family history of type 1 diabetes are associated with other autoimmune diseases, including coeliac disease, pernicious anaemia and thyroid disease. The peak incidence period differs for different autoimmune diseases. The graph shows the incidence of islet AAb (black line; associated with type 1 diabetes), transglutaminase (TG) AAb (red line; associated with coeliac disease), ATPase H^+^/K^+^ transporting subunit α (ATP4A) AAb (green line; associated with pernicious anaemia) and thyroid peroxidase (TPO) AAb (blue line; associated with thyroid disease), and their peak periods (horizontal bars). Adapted from [51], under the terms of the Creative Commons Attribution 4.0 International License (http://creativecommons.org/licenses/by/4.0/), which permits unrestricted use, distribution and reproduction in any medium. This figure is available as part of a downloadable slideset

**Fig. 2:**
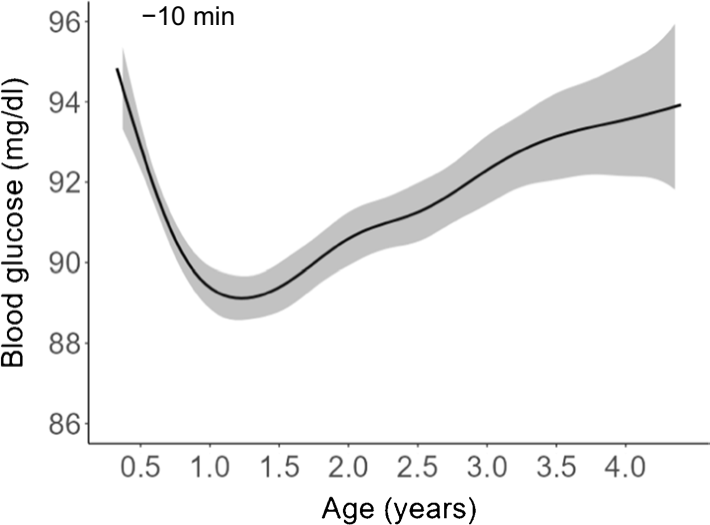
Preprandial blood glucose concentrations in relation to age in children at increased genetic risk for type 1 diabetes participating in the POInT study. Preprandial non-fasting glucose concentrations (10 min before food intake) were modelled using a general additive model with thin-plate splines based on 7925 measurements in *n*=1050 children. Adapted from [52], under the terms of the Creative Commons Attribution 4.0 International License (http://creativecommons.org/licenses/by/4.0/), which permits unrestricted use, distribution and reproduction in any medium. Data at 4 years previously unpublished (A. Weiss [Helmholtz Munich, German Center for Environmental Health, Munich, Germany], E. Bonifacio [Technische Universität Dresden, Dresden, Germany] and A.-G. Ziegler, unpublished results). To convert glucose to SI units (mmol/l), divide by 18. This figure is available as part of a downloadable slideset

Genes predisposing for type 1 diabetes and family history of type 1 diabetes are also associated with other autoimmune diseases, including coeliac disease, pernicious anaemia and thyroid disease [14, 51]. The incidence maxima of first-appearing autoantibodies is different for each of these autoimmune diseases. In individuals with a first-degree relative with type 1 diabetes, islet autoimmunity sharply increases first, followed by gut autoimmunity in coeliac disease and pernicious anaemia, peaking at 2 years of age, while thyroid autoimmunity peaks during puberty and adolescence (Fig. 1). This suggests that the affected organ is relevant, and its activity, function and maturity may change during the fertile period. So, why does beta cell autoimmunity occur so early in life? To indirectly investigate the changes in pancreatic islets with age, we measured random preprandial non-fasting blood glucose concentrations between 4 months and 3.5 years of age in children enrolled in POInT [52]. Intriguingly, there were substantial changes in glucose concentrations, and potentially pancreatic islet function, during the first year of life that inversely followed the incidence of islet autoimmunity (Fig. 2); notably, glucose concentration declined during the first year of life to a nadir at 1–1.5 years of age and increased after this age. This not only coincides with the peak incidence of islet autoimmunity but also follows the adiposity peak typically seen at 8–9 months of age, suggesting growth pressure on islets during the susceptible period. Thus, it is reasonable to propose that age 1 year may be a period of islet cell activity, increased beta cell stress and greater vulnerability to insults.

#### Exposures and immune responses

Respiratory infections are most frequent during the first 2 years of life [53, 54]. Children participating in the BABYDIAB/BABYDIET studies [53] were reported to have 3.7 infection episodes per 100 days during the peak period of islet autoimmunity (between 12 and 18 months of age). In the TEDDY study [54], 4–5 respiratory infection episodes were reported per person-year at ~1 year of age. The frequency declined by nearly half by 3–5 years of age. This observation is not unexpected because the protection provided by maternal antibodies against infection gradually declines while the child’s immune system develops following exposure to infection and vaccination. It is thought that while some viruses directly infect beta cells by entering through receptors, such as the coxsackie and adenovirus receptors (CXADRs), others may indirectly induce beta cell damage and immune activation via systemic inflammation.

**Fig. 3:**
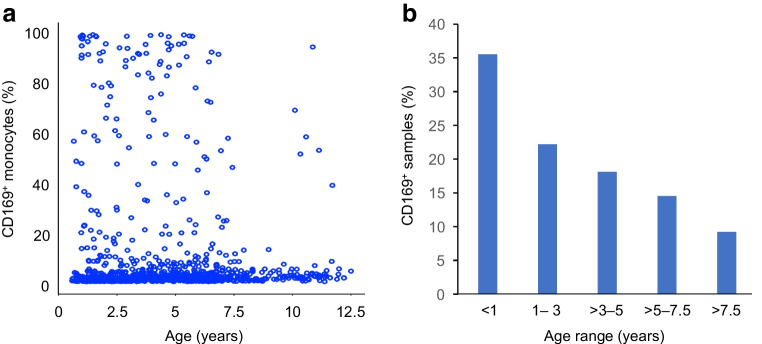
CD169 (sialic acid-binding immunoglobulin-like lectin 1 [SIGLEC-1]) expression in CD14^+^ monocytes in *n*=887 samples from *n*=262 children at increased risk for type 1 diabetes participating in prospective cohort studies. (**a**) Percentage of CD169^+^ cells in CD14^+^ monocytes in relation to age (regression coefficient: −0.20; *p*=2×10^−6^). A threshold of >5% positive monocytes was used to define monocyte CD169^+^ samples. (**b**) Monocyte CD169 expression by age group. Monocyte CD169 expression was more frequent in samples from younger children (*p*=0.0008, assessed by χ^2^ test), with a persistent decline with increasing age. Data in (**a**) and (**b**) are previously unpublished (E. Bonifacio [Technische Universität Dresden, Dresden, Germany] and A.-G. Ziegler, unpublished results). This figure is available as part of a downloadable slideset

Further evidence for frequent viral infection and a pronounced response to infection in the first years of life comes from studies of monocyte expression of CD169, which is transiently expressed in response to type 1 IFNs. We studied over 800 samples from children at increased risk for type 1 diabetes during the first 12 years of life and found that CD169 was expressed on monocytes in 35% of samples taken within the first year of life, declining to <10% after 7.5 years of age (Fig. 3; *p*=0.0008). This implies that the immune system is frequently challenged and responsive in early life. It is also possible that the responses are more intense and/or sustained in early life and that, overall, the body and its organs, as well as T cells, are frequently exposed to an inflammatory milieu. Indeed, inflammatory signatures and immune cells primed towards an inflammatory response are also found in children before they develop autoimmunity [17, 55, 56]. Supporting the notion that the inflammatory milieu fosters autoimmunity, CD4^+^ T cells are more likely to exhibit proinflammatory responses to insulin in vitro when the antigen is presented by CD169-expressing monocytes [41]. Moreover, children who subsequently developed islet autoantibodies had naive CD4^+^ T cells that reacted to the islet autoantigens proinsulin and GAD65 with a striking proinflammatory signature by 6 months of age, well before the appearance of memory islet autoantigen-responsive CD4^+^ T cells [17]. This suggests that children who develop islet autoimmunity have an a priori propensity to respond to beta cell autoantigens if presented. Consistent with these in vitro data, the BABYDIET study revealed an IFN-inducible transcriptional signature in peripheral blood mononuclear cells prior to seroconversion to the first islet autoantibody [55]. Upregulation of IFN-inducible genes was associated with a recent history of upper respiratory tract infections and marked by increased monocyte CD169 expression. Finally, the TEDDY study discovered a natural killer cell signature in children prior to the first islet autoantibody [56]. Taken together, these data derived from different studies and by using very different methods show that deviations from a healthy immune response with propensity to a T helper type 1 (Th1) response to insulin and marked inflammation are present before the onset of islet autoimmunity, and that these deviations could be a consequence of genetic priming and/or unfavourable responses to environmental insults.

### The case for a virus

Previous epidemiological and genetic data have associated viral infections with type 1 diabetes. Infections in the first year of life increase the risk of islet autoimmunity and type 1 diabetes [53, 54, 57]. The association is particularly high in children with prolonged or multiple respiratory infections. A large study of claims data showed that children with two or more infections by 6 months of age were more than twice as likely to develop type 1 diabetes by 8 years of age [58]. Furthermore, it was reported that children who develop islet autoimmunity experience their first viral infection earlier than children who do not develop islet autoimmunity or type 1 diabetes [57]. An increased frequency of viral infections in the time window before the first appearance of islet autoantibodies was also reported [53, 54]. This suggests that early childhood infections and infections shortly before the onset of autoimmunity play a role in promoting autoreactivity towards islet cells. Furthermore, multiple virus response genes have been linked to the risk of developing islet autoimmunity [59].

Repeated attempts have been made to identify which viruses are responsible for the increased risk of autoimmunity and diabetes. The candidates with most convincing evidence are enteroviruses, particularly coxsackie B virus. Evidence supporting this include an increased prevalence of coxsackie B virus infection prior to islet autoimmunity [60], the expression of CXADR on beta cells that provides an entry point for coxsackie B virus [61], and the presence of coxsackie B virus antigen in pancreases from people with islet autoantibodies or type 1 diabetes [62, 63]. Furthermore, the immune response to coxsackie B virus appears to be incomplete in young children with insulin autoimmunity [64]. Perhaps the strongest evidence for coxsackie B virus as a trigger of islet autoimmunity comes from the TEDDY study [65]. A large sequencing study of stool samples from more than 700 children suggested that prolonged shedding of enterovirus B*,* rather than short enterovirus B infections, may be involved in the development of islet autoimmunity [65]. However, only 11.8% of children with islet autoantibodies (vs 6.5% of children without islet autoantibodies) exhibited prolonged shedding of enterovirus B, suggesting that it is, at best, one of many aetiological causes of islet autoimmunity.

Studies have suggested the contributions of other viruses to the development of type 1 diabetes. For example, an increased incidence of type 1 diabetes was reported during the severe acute respiratory syndrome coronavirus 2 (SARS-CoV-2) pandemic [66, 67]. Similar to coxsackie B virus, the receptor for SARS-CoV-2 entry into cells, ACE2, is expressed on the pancreatic ductal cells, as well as on islet beta and alpha cells [68–70]; therefore, it is plausible that SARS-CoV-2 may trigger an autoimmune response to islets. However, screening of more than 50,000 youths in Colorado (USA) and Bavaria (Germany) suggested no association between SARS-CoV-2 infection and islet autoimmunity [71, 72]. It should be noted, however, that the cross-sectional design in this study did not allow the researchers to determine whether the autoantibodies developed before or after SARS-CoV-2 infection, and it remains unknown whether SARS-CoV-2 infection can lead to islet autoimmunity. Rotavirus and cytomegalovirus have also been implicated in islet autoimmunity [73–75], and a decrease in type 1 diabetes incidence was observed after the introduction of rotavirus vaccination in some, but not all studies [76–79]. The introduction of rotavirus vaccination has also hampered further investigations into the potential causal role of this virus.

Taken together, the available data corroborate that viruses, especially those capable of infecting islet cells in young children, are a ‘co-factor’ to genetics for the development of islet autoimmunity.

### Model of initiation during the susceptible/fertile period in early life

**Fig. 4:**
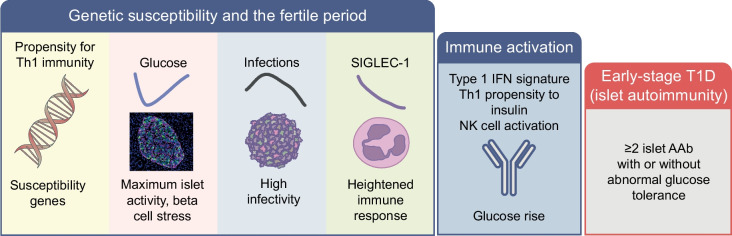
Model of initiation of islet autoimmunity. Genetic susceptibility and the fertile period around the age of peak islet autoantibody (AAb) incidence increase the likelihood of immune activation and sustained autoimmunity towards the beta cell (early-stage type 1 diabetes [T1D]). T1D susceptibility genes are predominantly immune-response genes and confer a propensity to Th1 immunity. It is proposed that early childhood is a fertile period for islet autoimmunity because of three factors that come together at this age: (1) high islet activity/stress, reflected by a nadir in glucose levels at 12–18 months of age (blue line); (2) high infectivity with a peak in viral infections (black curve); and (3) a heightened and then waning response by the immune system, as shown by the level and frequency of sialic acid-binding immunoglobulin-like lectin 1 (SIGLEC-1) expression on monocytes (purple line). Immune activation precedes islet autoimmunity as evidenced by type I IFN and natural killer (NK) cell signatures, proinflammatory T cell responses to insulin and a rise in glucose prior to islet AAb seroconversion. Seroconversion to ≥2 islet AAb without or with abnormal glucose tolerance defines early-stage T1D. Islet micrograph taken by T. Rodriguez-Calvo (Helmholtz Munich, Munich, Germany). This figure is available as part of a downloadable slideset

The countdown to type 1 diabetes starts early in life in children with genetic susceptibility. We have shown that several factors involving islets and the immune system join forces early in life to form an environment that might promote autoimmunity (Fig. 4). With regard to islets, their susceptibility for autoimmunity may be increased by modification of their activity at 12–18 months of age, indirectly shown by blood glucose concentrations and, which is likely to be amplified or attenuated by factors such as growth, weight and genetics. Increased islet activity could contribute to stress and alterations in the islet, exacerbating the effects of viral infections or other insults during this period. With regard to the immune system, a genetically determined Th1 propensity increases the likelihood that autoreactive cells are activated and expand in the inflammatory milieu owing, in part, to the many infections that occur in early childhood. For viral infections in the fertile period, either direct infection of the islet (with entry into beta cells) or general inflammation can alter the appearance of beta cells and increase antigen presentation, thereby unmasking the islets to the immune system [80].

One very recent piece of evidence supports the notion that an insult triggers beta cell autoimmunity. The current dogma hinges on metabolic deterioration subsequent to and resulting from the development of beta cell autoimmunity. In POInT, we investigated the temporal relationship between glucose levels and the development of islet autoantibodies. In contrast to the current dogma, we found that children who develop islet autoantibodies have raised glucose levels very early in the disease process that were evident prior to or concurrent with islet autoantibody seroconversion [52]. The rise in blood glucose levels shortly before islet autoantibody seroconversion is consistent with an event, such as a virus infection, that affects islet function and leads to the autoimmune response. Islet function continues to deteriorate after seroconversion, implying that pancreatic islet injury is a sustained (not transient) phenomenon. Other data suggest early endoplasmic reticulum stress within beta cells and reduced pancreas size/beta cell mass in people with islet autoantibodies and type 1 diabetes. Indeed, the pancreases of individuals with type 1 diabetes are smaller than those of control individuals, and danger signals, such as hyperexpression of HLA classes I and II, are present in pancreases of individuals with islet autoantibody-positivity [81–87]. Perhaps in the future, the utilisation of genetic risk scores and rapid point-of-care genetic testing will enable us to collect organ donor tissue from people with increased genetic risk of type 1 diabetes, without signs of islet autoimmunity or diabetes. Examining the pancreases of these donors could help clarify whether the changes previously observed in the islets of individuals at high risk of type 1 diabetes precede autoimmunity.

## Implications for prevention

What are the implications for primary prevention of type 1 diabetes? The Global Platform for the Prevention of Autoimmune Diabetes (GPPAD) was founded in 2017 to identify infants at elevated genetic risk of developing type 1 diabetes and to perform primary prevention trials aimed at reducing the incidence of islet autoimmunity and type 1 diabetes in children [88]. GPPAD uses a polygenic risk score to screen newborns for risk of type 1 diabetes in five European countries. The families of children with a polygenic risk score that identifies 1% of newborns and confers a >10% risk of developing multiple islet autoantibodies by 6 years of age are invited to participate in a randomised controlled trial of primary prevention of autoimmunity and type 1 diabetes [89]. Over 400,000 newborns have joined the GPPAD screening to date. GPPAD is currently conducting two randomised controlled trials. The first, POInT, aims to induce tolerance to insulin by oral insulin therapy and is examining whether oral insulin at increasing doses of up to 67.5 mg daily from 4 months to 3 years of age reduces the incidence of multiple islet autoantibodies vs placebo [8]. This therapy is intended to target the Th1 propensity to insulin in children with high genetic susceptibility. The enrolment of 1050 children has been completed; results are expected to be available in 2025. The second trial, Supplementation with *Bifidobacterium infantis* for Mitigation of Type 1 Diabetes Autoimmunity (SINT1A), aims to reduce inflammation and infection by promoting a healthy microbiome [90]. This trial is examining whether daily supplementation of *B. infantis* from 6 weeks to 12 months of age reduces the incidence of multiple islet autoantibodies vs placebo. The study is ongoing and over 600 infants of a target of 1150 have been enrolled to date (January 2023); results are expected to be available in 2027. Additional studies are also being planned by members of GPPAD. One goal of future studies would be to protect the beta cell from infection or stress to prevent the initiation of autoimmunity. Vaccination against coxsackie B or other viruses would be a desired strategy, and hopefully vaccinations will eventually become available.

The prevention of islet autoimmunity is a possible strategy to reduce the incidence of type 1 diabetes. Secondary prevention is another approach. Although preventive measures to halt type 1 diabetes progression are not discussed in this review, there has been an important breakthrough in preventive therapy for type 1 diabetes because the first disease-modifying therapy, teplizumab (also called TZIELD), was recently approved in the USA. Teplizumab can help ‘stop the clock’ and delay the progression from stage 2 to stage 3 type 1 diabetes by an average of 2 years. It is indicated for stage 2 type 1 diabetes (consisting of islet autoantibody positive and impaired glucose tolerance) and may be used in children with and without close relatives with type 1 diabetes. This new therapy makes screening programmes for islet autoantibodies a necessity to identify all children who might benefit from teplizumab. Excitingly, it has opened up a new era, moving closer towards a world without type 1 diabetes.

## Supplementary Information

Below is the link to the electronic supplementary material.

Supplementary file1 (PPTX 576 KB)

## Data Availability

Data will be available with a signed transfer agreement; please contact the corresponding author.

## Abbreviations

CXADR: Coxsackie and adenovirus receptors
GPPAD: Global Platform for the Prevention of Autoimmune Diabetes
POInT: Primary Oral Insulin Trial
SARS-CoV-2: Severe acute respiratory syndrome coronavirus 2
TEDDY: The Environmental Determinants of Diabetes in the Young
Th1: T helper type 1
Treg: Regulatory T cells
