# Supplementary material for: The countdown to type 1 diabetes: when, how and why does the clock start?
Source: Diabetologia. 2023 May 26;66(7):1169–78. doi: 10.1007/s00125-023-05927-2 (PMC10212739; doi:10.1007/s00125-023-05927-2)
Supplement: Supplementary file 1 — Supplementary file1 (PPTX 576 KB) [file 125_2023_5927_MOESM1_ESM.pptx]

## Slide 1
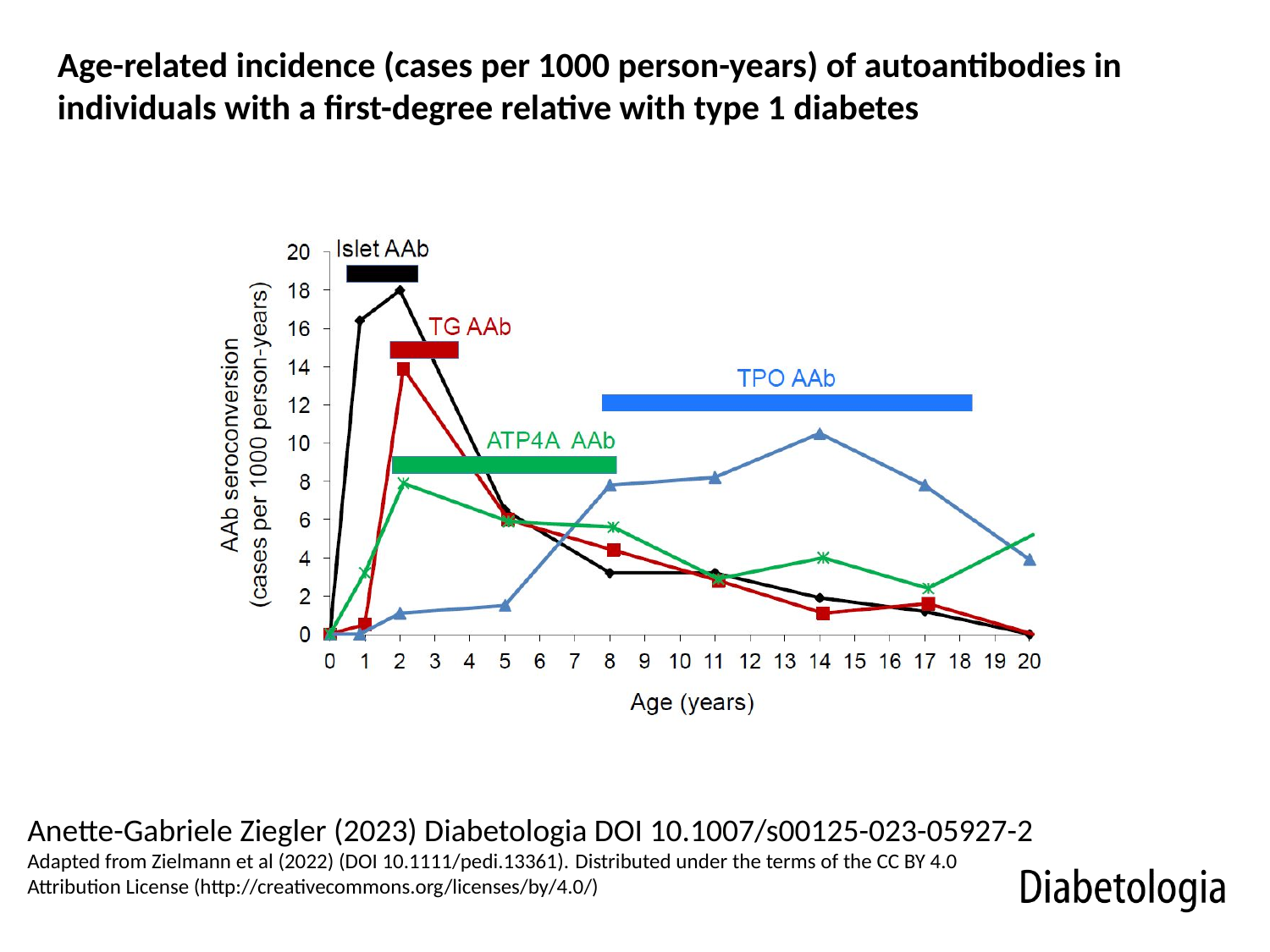

Age-related incidence (cases per 1000 person-years) of autoantibodies in individuals with a first-degree relative with type 1 diabetes
Anette-Gabriele Ziegler (2023) Diabetologia DOI 10.1007/s00125-023-05927-2
Adapted from Zielmann et al (2022) (DOI 10.1111/pedi.13361). Distributed under the terms of the CC BY 4.0
Attribution License (http://creativecommons.org/licenses/by/4.0/)

## Slide 2
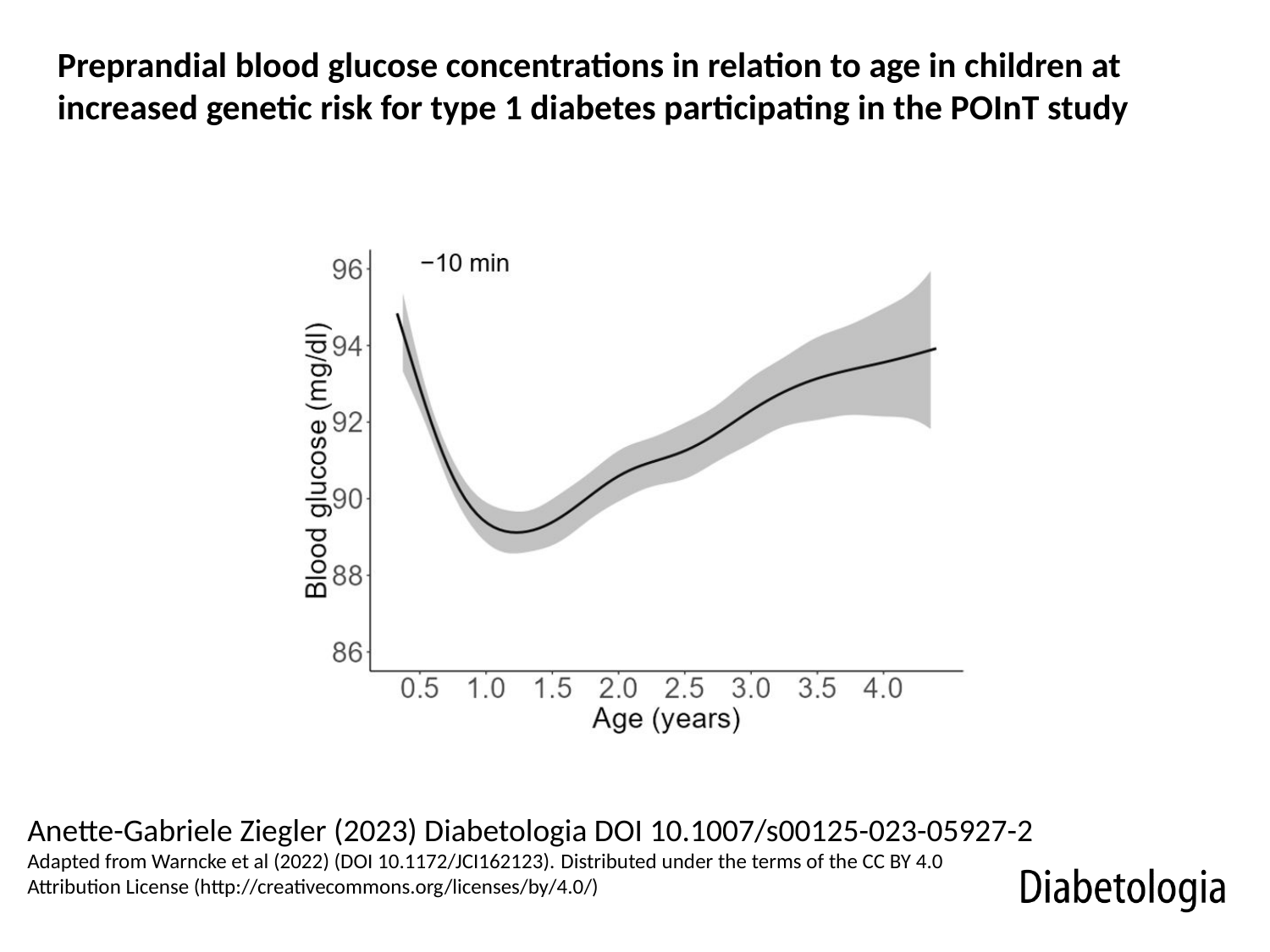

Preprandial blood glucose concentrations in relation to age in children at increased genetic risk for type 1 diabetes participating in the POInT study
Anette-Gabriele Ziegler (2023) Diabetologia DOI 10.1007/s00125-023-05927-2
Adapted from Warncke et al (2022) (DOI 10.1172/JCI162123). Distributed under the terms of the CC BY 4.0
Attribution License (http://creativecommons.org/licenses/by/4.0/)

## Slide 3
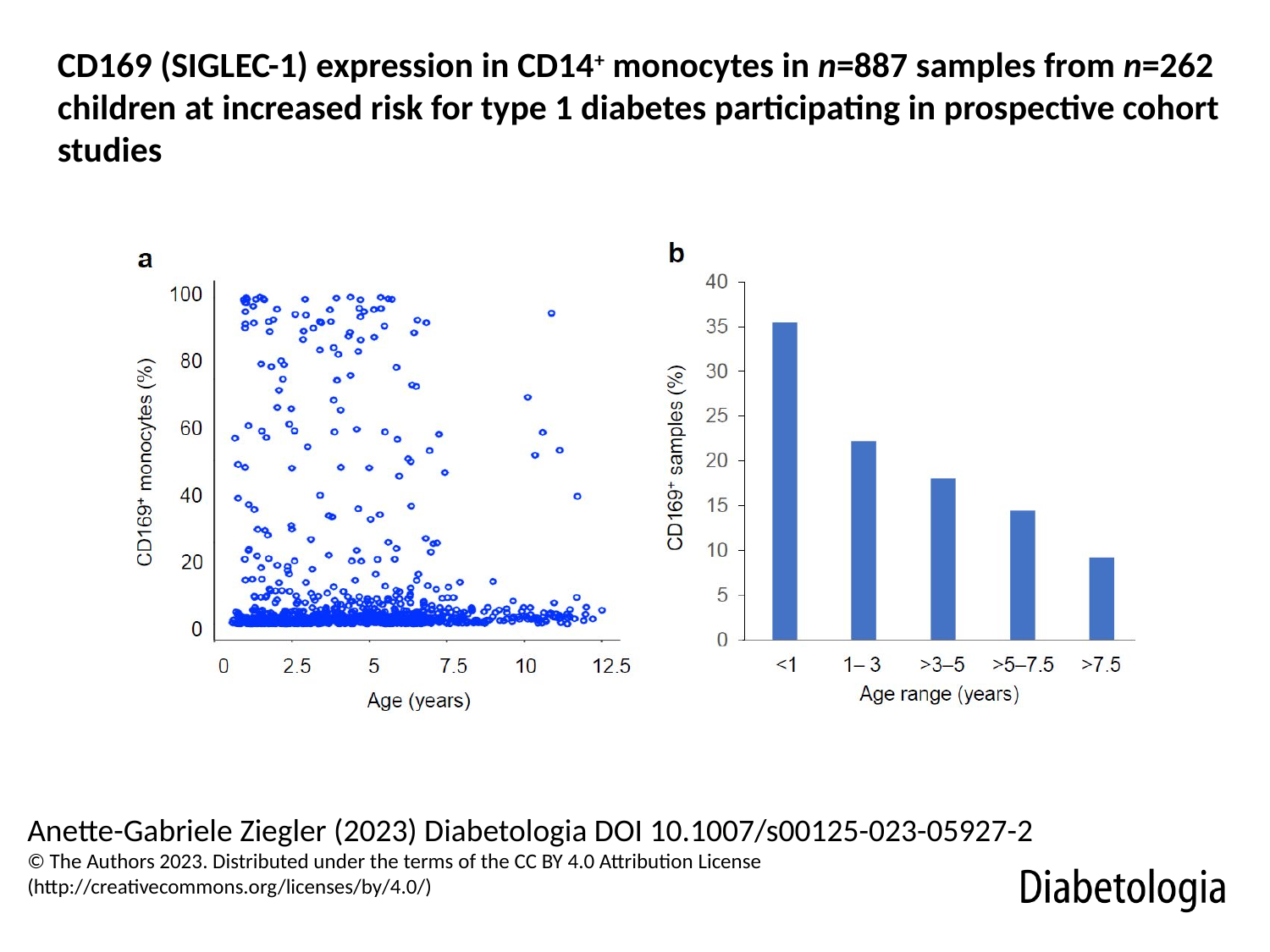

CD169 (SIGLEC-1) expression in CD14+ monocytes in n=887 samples from n=262 children at increased risk for type 1 diabetes participating in prospective cohort studies
Anette-Gabriele Ziegler (2023) Diabetologia DOI 10.1007/s00125-023-05927-2
© The Authors 2023. Distributed under the terms of the CC BY 4.0 Attribution License (http://creativecommons.org/licenses/by/4.0/)

## Slide 4
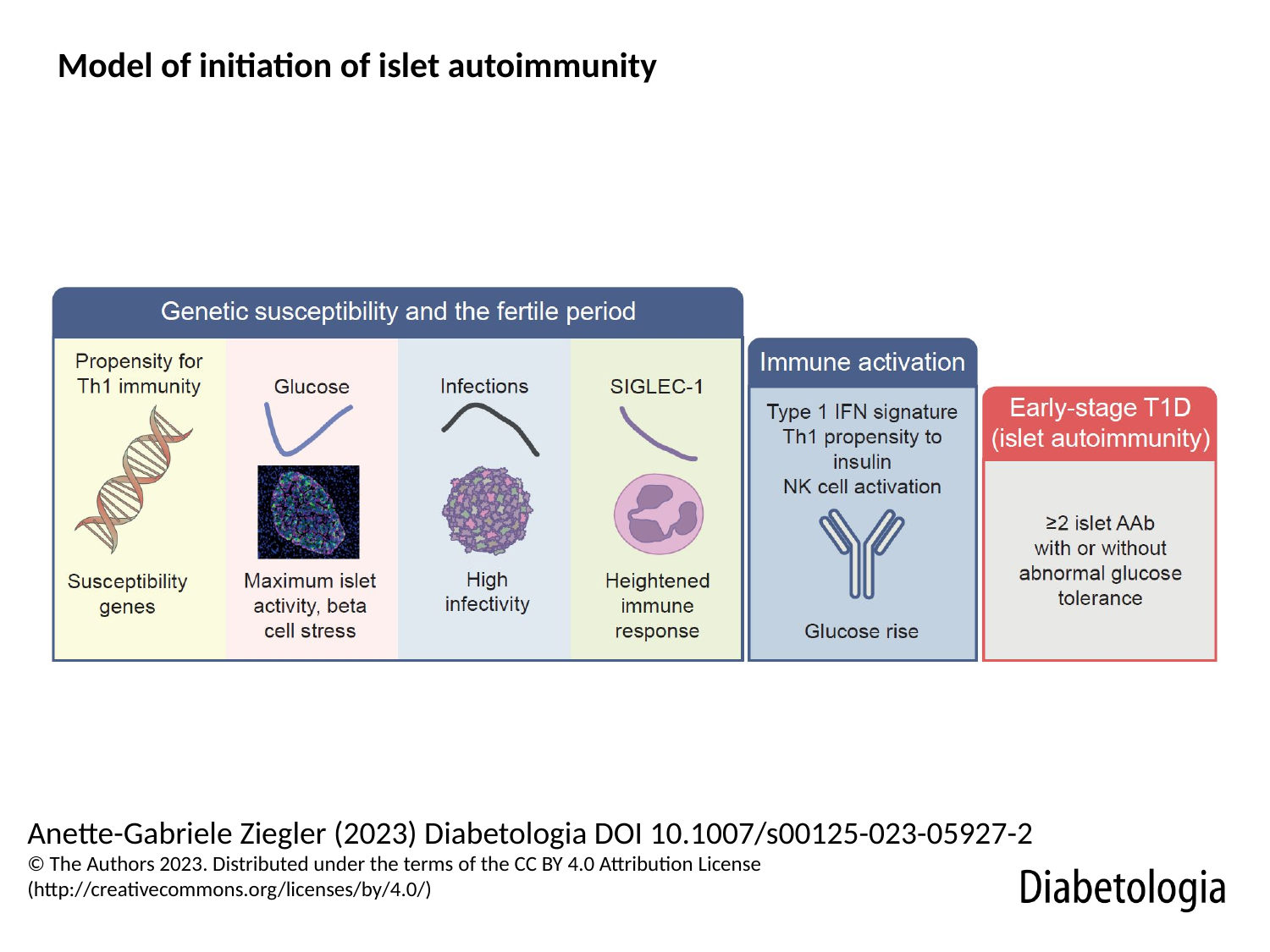

Model of initiation of islet autoimmunity
Anette-Gabriele Ziegler (2023) Diabetologia DOI 10.1007/s00125-023-05927-2
© The Authors 2023. Distributed under the terms of the CC BY 4.0 Attribution License (http://creativecommons.org/licenses/by/4.0/)
